# Immune response to vaccination against SARS-CoV-2 in hematopoietic stem cell transplantation and CAR T-cell therapy recipients

**DOI:** 10.1186/s13045-022-01300-9

**Published:** 2022-06-16

**Authors:** Xi Wu, Lu Wang, Lu Shen, Lin He, Kefu Tang

**Affiliations:** 1 Bio-X Institutes, Key Laboratory for the Genetics of Developmental and Neuropsychiatric Disorders (Ministry of Education), Shanghai Jiao Tong University, Shanghai, 200030 China; 2 Prenatal Diagnosis Center, Department of Clinical Laboratory, Changning Maternity and Infant Health Hospital, East China Normal University, Shanghai, 200051 China

**Keywords:** Hematopoietic stem cell transplantation (HSCT), Chimeric antigen receptor T-cell (CAR-T) therapy, SARS-CoV-2 vaccination, Immune response

## Abstract

Recipients after hematopoietic stem cell transplantation (HSCT) or chimeric antigen receptor T-cell (CAR-T) therapy are at increased risk for unfavorable outcomes after SARS-CoV-2 infection. The efficacy of COVID-19 vaccines remains undetermined in this vulnerable population, we therefore conducted a pooled analysis to evaluate the immune response after vaccination. A total of 46 studies were finally included, comprising 4757 HSCT and 174 CAR-T recipients. Our results indicated that HSCT and CAR-T recipients had an attenuated immune response to SARS-CoV-2 vaccination compared with healthy individuals, while time interval between transplant and vaccination, immunosuppressive therapy (IST) and lymphocyte counts at vaccination significantly affected the humoral response in HSCT recipients. In addition, seroconversion was significantly higher in patients with BCMA-based CAR-T than those with CD19-based CAR-T. Thus, an adapted vaccination strategy for HSCT and CAR-T recipients may be required, and further research on the effect of a booster dose of COVID-19 vaccine and the role of cellular response after vaccination is warranted.

**Supplementary Information:**

The online version contains supplementary material available at 10.1186/s13045-022-01300-9.

To the Editor,

The pandemic caused by SARS-CoV-2 has led to global mortality of over 6 million deaths and vaccination is the primary strategy to stop this public health emergency. Recipients after hematopoietic stem cell transplantation (HSCT) or chimeric antigen receptor T-cell (CAR-T) therapy are at increased risk for severe COVID-19 and unfavorable outcomes [1]. Previous studies showed blunted humoral responses to vaccination against SARS-CoV-2 among HSCT and CAR-T recipients [2–6]. With emerging evidence available, we performed a comprehensive meta-analysis to evaluate the immune responses to COVID-19 vaccines in recipients of HSCT and CAR-T (Additional file 1: Methods S1 and Additional file 1: Fig. S1).

Overall, 44 studies comprising 4757 HSCT patients [1182 of autologous HSCT (autoHSCT), 3495 of allogeneic HSCT (alloHSCT), 80 of autoHSCT or alloHSCT (mixed)] (Additional file 1: Table S1) and 12 studies comprising 174 CAR-T recipients were included (Additional file 1: Table S2). For HSCT, five studies investigated the response after partial vaccination, 38 studies evaluated the response after completed vaccination, and seven studies assessed the response after a third dose. As for CAR-T, 11 studies evaluated the response after completed vaccination, and one study assessed the response following a third dose.

The seropositive rates of the second and third dose were significantly higher than the first dose, while no significant difference in seroconversion between the second and third dose (Fig. 1A). The pooled humoral response rate was 81.4% following completed vaccination in HSCT patients (Additional file 1: Fig. S2), with response rates of 86.1% and 79.6% for autoHSCT and alloHSCT. The response rates after one and three vaccine doses were 40.8% and 78.6%, respectively (Additional file 1: Figs. S3–S4). Although pooled analysis could not be performed due to heterogeneity of data, significantly lower antibody titres were observed in HSCT patients compared with healthy controls (Additional file 1: Table S3).

**Fig. 1:**
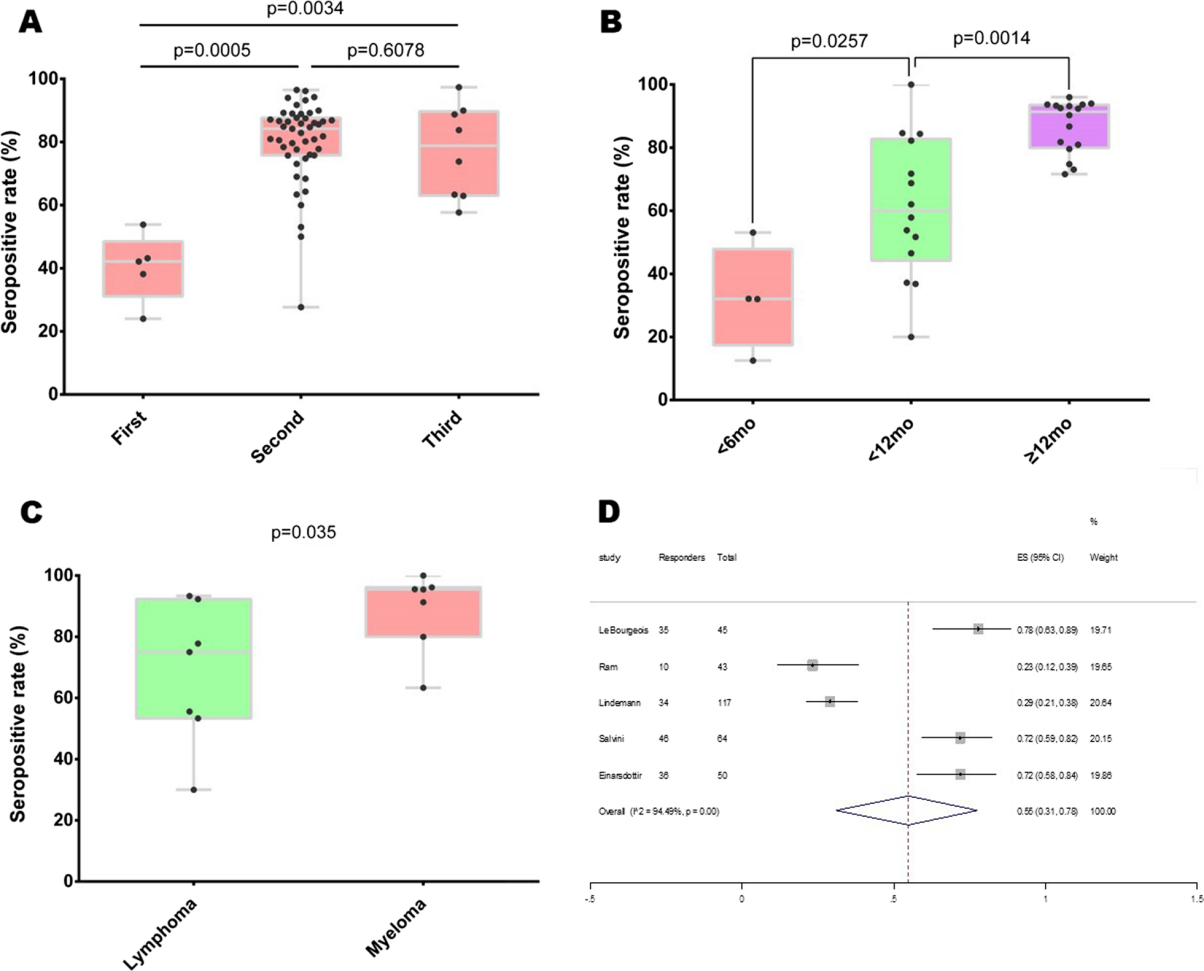
The immune response to SARS-CoV-2 vaccines in HSCT recipients. **A** Boxplots of seropositive rates (%) after the first, second and third dose of vaccination; **B** boxplots of seropositive rates (%) according to the interval between transplant and vaccination (< 6 months, between 6 and 12 months and ≥ 12 months); **C** boxplots of seropositive rates (%) in autoHSCT recipients according to underlying diseases (lymphoma or myeloma); **D** pooled analysis of T-cell response rate based on IFN-γ ELISPOT assay after vaccination. In boxplots, each point indicates a study cohort where data were available. Pairwise comparisons are based on the nonparametric Mann–Whitney U independent-samples test

Our results indicated response rate significantly increased with the time interval from HSCT to vaccination: 38.2% within 6 months, 62.3% between 6 and 12 months, and 87.9% after 12 months (Fig. 1B and Additional file 1: Fig. S5). Among autoHSCT recipients, stratified analysis by underlying diseases demonstrated myeloma patients had a marginal increased seroconversion rate compared to lymphoma patients (*P* = 0.035, Fig. 1C).

In addition, immunosuppressive therapy (IST: OR = 5.86, 95% CI: 3.74–9.18, *P* < 10^−5^; Additional file 1: Fig. S6) and lymphopenia (lymphocyte counts < 1G/L among alloHSCT: OR = 4.44, 95% CI: 2.56–7.70, *P* < 10^–5^; Additional file 1: Fig. S7) at vaccination were significantly associated with seronegative response. And neither the status of graft-versus-host disease (GVHD) (Additional file 1: Fig. S8) nor age (Additional file 1: Fig. S9) was significantly associated with seroconversion after vaccination. Furthermore, T-cell response rate based on IFN-γ ELISPOT assay was 55% in HSCT patients (Fig. 1D).

As for CAR-T recipients, the combined serological response rate after SARS-CoV-2 vaccination was 35.9% (Fig. 2A). When stratified by different constructs, seroconversion was significantly higher in patients with BCMA-based CAR-T than those with CD19-based CAR-T (Fig. 2B).

**Fig. 2:**
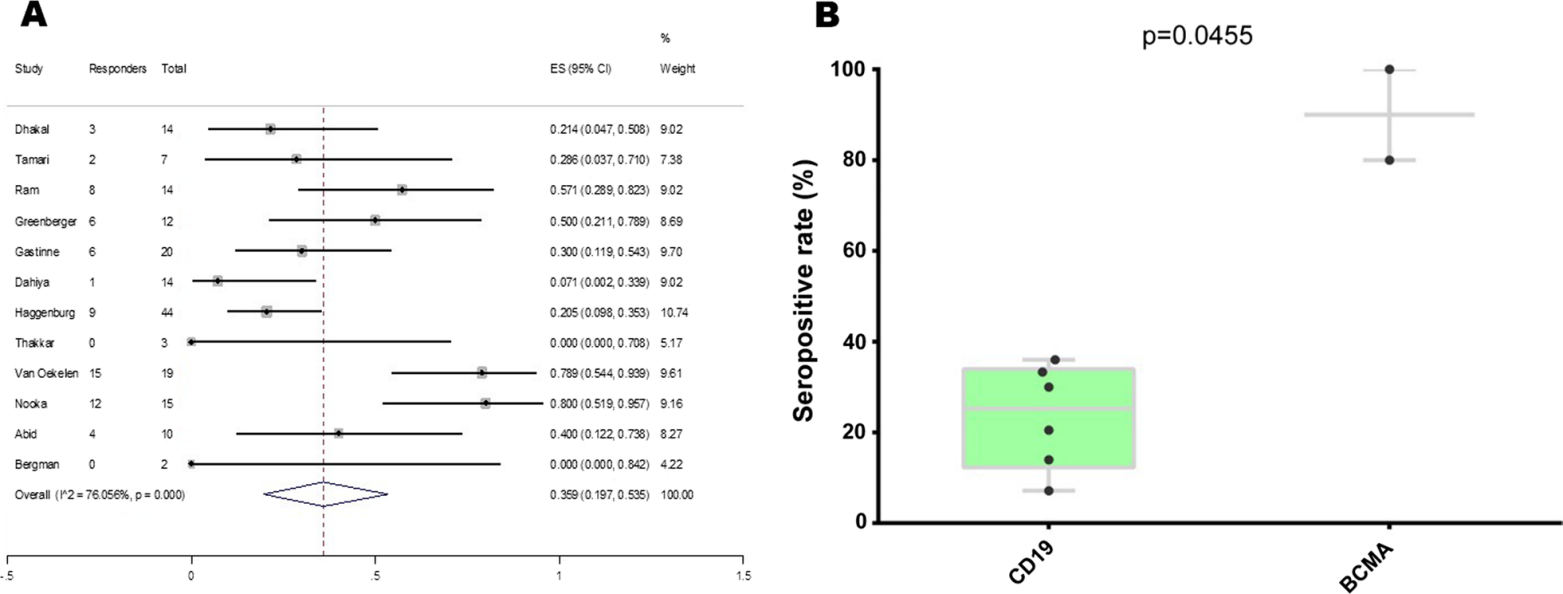
The serological response to SARS-CoV-2 vaccines in CAR-T recipients. **A** Pooled analysis of serological response rate after vaccination; **B** boxplots of seropositive rates (%) according to CAR-T constructs (CD19 and BCMA). Each point indicates a study cohort where data were available. Pairwise comparisons are based on the nonparametric Mann–Whitney U independent-samples test

Our analysis demonstrated suboptimal immune responses to SARS-CoV-2 vaccination in patients after HSCT and CAR-T, especially for CD19-based CAR-T recipients. Although we found no significant difference between the second and third dose, the boost vaccination against SARS-CoV-2 was identified to improve humoral response in HSCT patients initially seronegative following the second dose [7, 8]. Moreover, antibody levels were reported to significantly increase after the third dose, counteracting the waning immunity after completed vaccination [9, 10]. Of note, new evidence illustrated a majority of alloHSCT patients without GVHD produced neutralizing antibody against Delta and Omicron variants after a third dose [11], underscoring the benefits of a booster dose. Additionally, we found HSCT patients vaccinated after recent transplantation, on IST or with lymphopenia were at higher risk of insufficient responses to COVID-19 vaccines, indicating the importance of immune recovery status for SARS-CoV-2 vaccination.

Interestingly, seroconversion rate was significantly higher in patients with BCMA-based CAR-T compared to those with CD19-directed CAR-T. But due to sparse data, more studies are needed to validate our result and further investigate the impact of different CAR-T constructs on serological response after SARS-CoV-2 vaccination.

In summary, our study indicated that HSCT and CAR-T recipients developed impaired immune responses to COVID-19 vaccines. Thus, an adapted vaccination strategy for these patients may be required. Moreover, the effect of a booster dose and the role of cellular response after SARS-CoV-2 vaccination in HSCT and CAR-T recipients should be addressed in future research.

## Supplementary Information

**Additional file 1:** Methods S1. **Supplementary Figure S1.** Flowchart of study selection. **Supplementary Figure S2.** Forest plots for the pooled analysis of serological response after completed vaccination in HSCT recipients. **Supplementary Figure S3.** Forest plots for the pooled analysis of serological response after one dose of vaccination in HSCT recipients. **Supplementary Figure S4.** Forest plots for the pooled analysis of serological response after three doses of vaccination in HSCT recipients. **Supplementary Figure S5.** Forest plots for the pooled analysis of serological response according to the interval between transplant and vaccination (<6 months, between 6-12 months and ≥12 months). **Supplementary Figure S6.** Forest plots for the association of immunosuppressive therapy and the risk of seronegative response after COVID-19 vaccination in HSCT recipients. **Supplementary Figure S7.** Forest plots for the association of lymphopenia and the risk of seronegative response after COVID-19 vaccination in alloHSCT recipients. **Supplementary Figure S8.** Forest plots for the association of the status of GVHD and the risk of seronegative response after COVID-19 vaccination in HSCT recipients. **Supplementary Figure S9.** Forest plots for the association of age and the risk of seronegative response after COVID-19 vaccination in HSCT recipients. **Supplementary Table S1.** Study characteristics for HSCT. **Supplementary Table S2.** Study characteristics for CAR-T. **Supplemental Table S3.** Anti-Spike (S) SARS-CoV-2 antibody titres in HSCT patients vs. healthy controls.

## Data Availability

The datasets used and/or analyzed during the current study are available from the corresponding author on reasonable request.

## Abbreviations

HSCT: Hematopoietic stem cell transplantation
CAR-T: Chimeric antigen receptor T-cell
SARS-CoV-2: Severe acute respiratory syndrome coronavirus 2
COVID-19: Coronavirus disease 2019
autoHSCT: Autologous HSCT
alloHSCT: Allogeneic HSCT
IFN-γ: Interferon-gamma
ELISPOT: Enzyme-linked immunosorbent spot
IST: Immunosuppressive therapy
OR: Odds ratio
CIs: Confidence intervals
GVHD: Graft-versus-host disease
