# Supplementary material for: Immune response to vaccination against SARS-CoV-2 in hematopoietic stem cell transplantation and CAR T-cell therapy recipients
Source: J Hematol Oncol. 2022 Jun 16;15:81. doi: 10.1186/s13045-022-01300-9 (PMC9200932; doi:10.1186/s13045-022-01300-9)
Supplement: Supplementary file 1 — Additional file 1: Methods S1. Supplementary Figure S1. Flowchart of study selection. Supplementary Figure S2. Forest plots for the pooled analysis of serological response after completed vaccination in HSCT recipients. Supplementary Figure S3. Forest plots for the pooled analysis of serological response after one dose of vaccination in HSCT recipients. Supplementary Figure S4. Forest plots for the pooled analysis of serological response after three doses of vaccination in HSCT recipients. Supplementary Figure S5. Forest plots for the pooled analysis of serological response according to the interval between transplant and vaccination (<6 months, between 6-12 months and ≥12 months). Supplementary Figure S6. Forest plots for the association of immunosuppressive therapy and the risk of seronegative response after COVID-19 vaccination in HSCT recipients. Supplementary Figure S7. Forest plots for the association of lymphopenia and the risk of seronegative response after COVID-19 vaccination in alloHSCT recipients. Supplementary Figure S8. Forest plots for the association of the status of GVHD and the risk of seronegative response after COVID-19 vaccination in HSCT recipients. Supplementary Figure S9. Forest plots for the association of age and the risk of seronegative response after COVID-19 vaccination in HSCT recipients. Supplementary Table S1. Study characteristics for HSCT. Supplementary Table S2. Study characteristics for CAR-T. Supplemental Table S3. Anti-Spike (S) SARS-CoV-2 antibody titres in HSCT patients vs. healthy controls. [file 13045_2022_1300_MOESM1_ESM.docx]

**Supplementary Materials**

**Methods S1**

The PRISMA guidelines were followed in the present study (Figure S1). Literature search from December 1, 2019 to April 1, 2022 was conducted in PubMed, Embase and Cochrane Library without language restriction. The search term included keywords relevant to “COVID-19”, “SARS-CoV-2”, “vaccine”, “vaccination”, “hematopoietic stem cell transplantation (HSCT)” and “chimeric antigen receptor T-cell (CAR-T)”.

**Inclusion** **criteria**

Eligible studies were required to meet the following criteria: (1) clinical study evaluating anti-S IgG or T-cell responses in HSCT or CAR-T recipients after vaccination against SARS-CoV-2; (2) original articles reporting independent data; (3) reporting the humoral or cellular response rates to SARS-CoV-2 vaccination, or providing sufficient information for calculating the response rate or odds ratio (OR) (2 x 2 contingency table is required for OR calculation, and the numbers of patients with positive antibody or T-cell responses and total patients are required for response rate calculation). There is no limitation of the types of SARS-CoV-2 vaccines. Receiving one dose of mRNA vaccine was defined as partial vaccination, completing two doses of mRNA/inactivated vaccines or one dose of adenoviral vaccine was defined as completed vaccination, and a third dose was defined as a booster dose. The titles and abstracts of potential articles were screened by two authors independently, and the full-texts of potentially relevant articles were assessed. The references of included studies were scrutinized and hand-searched for additional eligible studies.

**Exclusion** **criteria**

The exclusion criteria were commentaries, reviews, non-research letters, case reports, and studies with overlapping samples or insufficient data for calculation of the response rate or ORs with 95% confidence intervals (CIs). One study was excluded which reported one with positive response among three CAR-T patients, without testing response of the other two (Lim et al, 2021).

**Outcomes**

The primary outcome was to assess the proportion of patients after HSCT and CAR-T therapy classified as vaccine responders (number of patients with anti-S SARS-CoV-2 IgG levels above each individual study’s cut-off value versus all patients). The secondary outcome was the proportion of patients with positive T-cell response (evaluated by ELISpot tests) in HSCT and CAR-T recipients after COVID-19 vaccination.

**Data extraction**

The following variables were recorded: authorship, publication year, country, study design, numbers of patients, age, sex, type of HSCT (autologous or allogeneic), number of patients with response to vaccination, underlying disease, type and number of COVID-19 vaccine doses, type of anti-S IgG immunoassay and cut-off value used to deﬁne serological response, time from HSCT or CAR-T therapy to vaccination.

**Statistical analysis**

Response rate to COVID-19 vaccines in patients after HSCT or CAR-T therapy, with corresponding 95% CIs calculated and pooled using the random-effects method. The effect size for binary outcomes was presented as ORs. Serological responses were separately assessed after first, second or third dose of vaccine. Cochran's Q test and I^2^ index (≤50% as low, 50%-75% as moderate, >75% as high) were calculated to explore heterogeneity across studies. Subgroup analyses were performed based on type of HSCT [recipients of autologous HSCT (autoHSCT group), recipients of allogeneic HSCT (alloHSCT group), and recipients with autoHSCT or alloHSCT (defined as mixed group)], time interval from HSCT to vaccination, underlying diseases among autoHSCT recipients, age, the status of graft-versus-host disease (GVHD), lymphocyte counts and immunosuppressive therapy (IST) at vaccination. Sensitivity analysis was performed to investigate whether individual study influenced the pooled result by removing one study at a time and reanalyzing the remaining studies. Type I error rate was set at 0.05 for two-sided analysis. All statistical analyses were done using the STATA software (version 14.0).


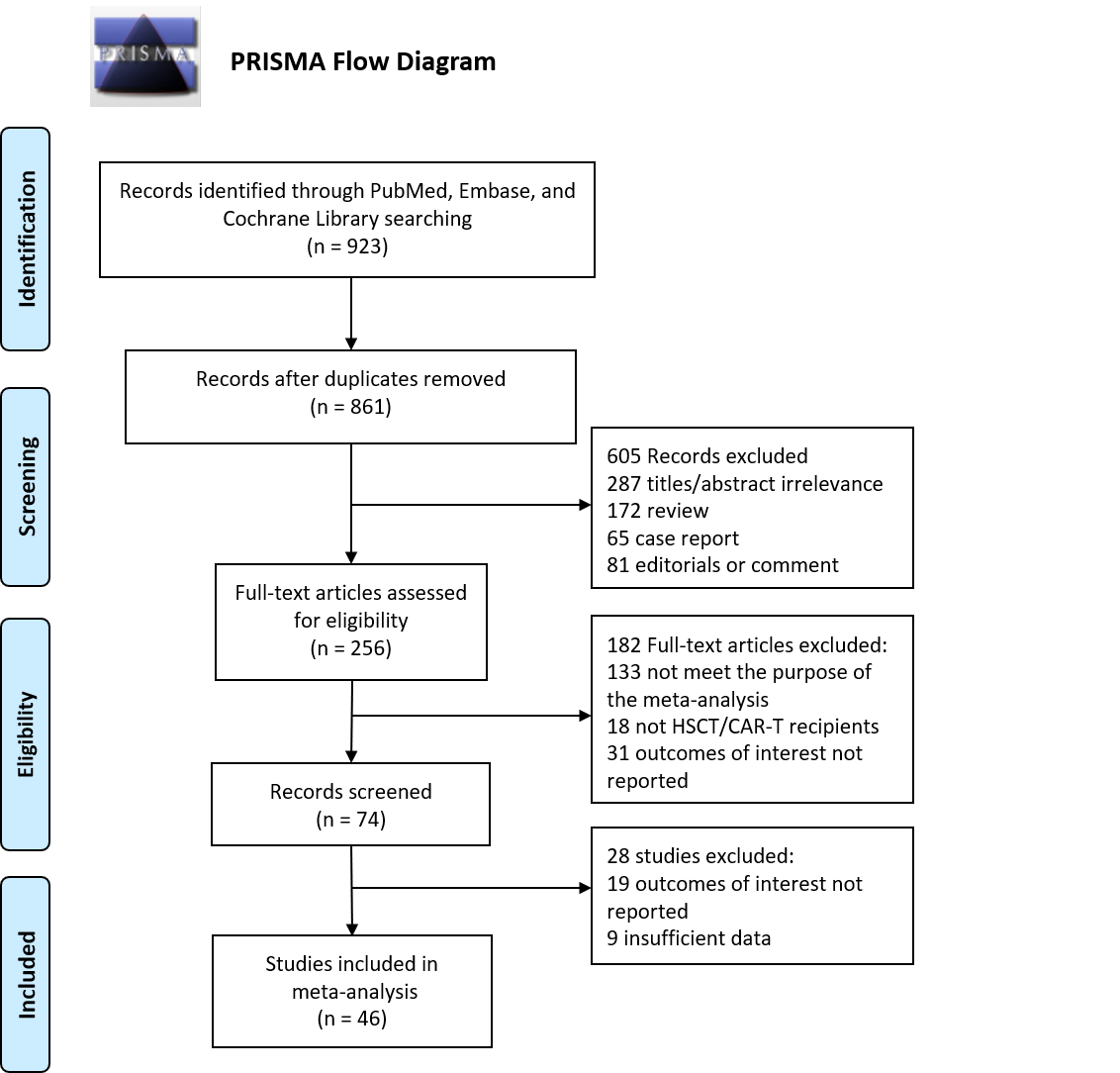


**Supplementary Figure S1. Flowchart of study selection.**

**Supplementary Figure S2. Forest plots for the pooled analysis of serological response after completed vaccination in HSCT recipients.**

**Supplementary Figure S3. Forest plots for the pooled analysis of serological response after one dose of vaccination in HSCT recipients.**

**Supplementary Figure S4. Forest plots for the pooled analysis of serological response after three doses of vaccination in HSCT recipients.**

**Supplementary Figure S5. Forest plots for the pooled analysis of serological response according to the interval between transplant and vaccination (<6 months, between 6-12 months and ≥12 months).**

**Supplementary Figure S6. Forest plots for the association of immunosuppressive therapy and** **the risk of seronegative response after COVID-19 vaccination in HSCT recipients.**

**Supplementary Figure S7. Forest plots for the association of** **lymphopenia and the risk of seronegative response after COVID-19 vaccination in alloHSCT recipients.**

**Supplementary Figure S8. Forest plots for the association of** **the status of GVHD and the risk of seronegative response after COVID-19 vaccination in HSCT recipients.**

**Supplementary Figure S9. Forest plots for the association of age and the risk of seronegative response after COVID-19 vaccination in HSCT recipients.**

**Supplementary Table S1. Study characteristics for HSCT.**

| Study | Country | Study design | No. of patients | Vaccine type | Dosage | Test assay | Cut-off | Disease | Median age (years) | Gender (male %) | Median time for vaccination after HSCT (months) | Internal between the vaccine and response assessment |
| --- | --- | --- | --- | --- | --- | --- | --- | --- | --- | --- | --- | --- |
| Herzog Tzarfati [1] | Israel | NA | 21(autologous) | BNT162b2 | 2nd | DiaSorin | 12 AU/ml | NA | NA | NA | NA | 32 days (IQR 29–40) after the second dose |
| Avivi [2] | Israel | prospective cohort study | 96(autologous) | BNT162b2 | 2nd | Roche | 0.8 U/ml | multiple myeloma | NA | NA | 36 (IQR 20–56) | 14–21 days after the second dose |
| Dhakal [3] | USA | NA | 45(autologous)+71(allogeneic) | BNT162b2, mRNA-1273, Ad26.COV2.S | 2nd or 1st | Euroimmun ELISA | 1.1 | NA | autoHSCT: 65 (seropositive and seronegative patients); alloHSCT: 64 (seropositive patients) and 68.5 (seronegative patients) | NA | autoHSCT: 30 (seropositive patients) and 30 (seronegative patients); alloHSCT: 26 (seropositive patients) and 25 (seronegative patients) | ≥ 2weeks after completed vaccination |
| Tamari [4] | USA | prospective observational study | 61(autologous)+149(allogeneic) | BNT162b2, mRNA-1273 | 2nd | Abbott | 50.0 AU/mL | NA | 66.4 (25.8–84.1)* | 59.4%* | 33.6 days (IQR 16.3-58.7)* | about 2 months after the second dose |
| Le Bourgeois [5] | France | observational study | 117(allogeneic) | BNT162b2 | 1st + 2nd | Roche | 0.8 U/ml | myeloma, lymphoma | 57 (range 20-75) | 60% | NA | at the time of second dose and about 1 month after second dose |
| Maneikis [6] | Lithuania | prospective cohort study | 192(autologous)+122(allogeneic) | BNT162b2 | 2nd | Abbott | 50 AU/mL | ALL, AML, HLH, CML, MDS, AA, MM, FL, DLBCL, CLL, MCL, T-NHL, HL, B-NHL | AutoHSCT: 63 (range 54–69); alloHSCT: 55 (range 43–65) | AutoHSCT: 45%; alloHSCT: 50% | NA | 7–21 days after the second dose |
| Easdale [7] | UK | retrospective study | 55(allogeneic) | BNT162b2, AZD1222 | 1st | Ortho | 1 s/c | ALL, AML, AA, MDS, NHL, HL, MF | 50 (range 18-73) | 62% | 15 (range 3.6-151.1) | 42.1 days (range 14-84) after the first dose |
| Redjoul [8] | France | NA | 88(allogeneic) | BNT162b2 | 2nd | Abbott | 50.0 AU/mL | NA | NA | NA | 23 (range 3–213) | 28 days (IQR 26–31) after the second dose |
| Ram [9] | Israel | prospective cohort study | 66(allogeneic) | BNT162b2 | 2nd | Roche | 0.8 U/ml | NA | NA | NA | 32 (range 3-263) | 7-14 days after the second dose |
| Greenberger [10] | USA | prospective cohort study | 73(allogeneic) | BNT162b2, mRNA-1273 | 2nd | Roche | 0.8 U/ml | NA | NA | NA | NA | 41 days (range 14, 134) after the second dose |
| Thakkar [11] | USA | cross-sectional cohort study | 26(Mixed) | BNT162b2, mRNA-1273, Ad26.COV2.S | 2nd or 1st | Abbott | 50.0 AU/mL | NA | NA | NA | NA | at least 7 days after completed vaccination |
| Van Oekelen [12] | USA | NA | 132(autologous) | BNT162b2, mRNA-1273 | 2nd | Kantaro ELISA | 5 AU/mL | MM | NA | NA | NA | 51 days (range 11–118) after the second dose |
| Ehmsen [13] | Denmark | observational prospective cohort study | 54(Mixed) | BNT162b2, mRNA-1273 | 2nd | Abbott | 50.0 AU/mL | NA | NA | NA | NA | 36 days (IQR 29–43) after the second dose |
| Shem-Tov [14] | Israel | NA | 152(allogeneic) | BNT162b2 | 2nd | Euroimmun ELISA | 1.1 | AML, MDS, NHL, HL, CLL, MPD, MF, CML, ALL, AA | 58 (range 22–82) | 63% | 41 (IQR 24-76) | 28 days (IQR 8–69) after the second dose |
| Attolico [15] | Italy | prospective cohort study | 52(autologous)+62(allogeneic) | BNT162b2 | 2nd | Abbott | 50.0 AU/mL | AML, ALL, SAA, CML, MDS, NHL, MM, CMML, MF, CLL, HL | AlloHSCT: 56 (range 28–70); AutoHSCT: 57 (range 20–71) | 57% | NA | 7–21 days after the second dose |
| Le Bourgeois [16] | France | retrospective study | 80(allogeneic) | BNT162b2 | 3rd | Roche | 0.8 U/ml | Myeloid, Lymphoid, and others | 57 (range 20–75) | 56% | 24 (range 3–207) | NA |
| Lindemann [17] | Germany | NA | 117(allogeneic) | BNT162b2, BNT162b2, AZD1222 | 2nd or 1st | Euroimmun ELISA | 1.1 | Acute leukemia, MDS, MPN, Lymphoma and others | 59 (range 21–77) | 48% | 30 (range 5–391) | 31 days (range 11–137) |
| Matkowska-Kocjan [18] | Poland | prospective observative study | 57(allogeneic) | BNT162b2 | 2nd | Euroimmun/PerkinElmer ELISA | 35.2 BAU/mL | ALL, SAA, CML, MDS, AML, Fanconi Anemia, severe combined immunodeficiency, common variable immunodeficiency, primitive neuroectodermal tumor, metachromatic leukodystrophy, Blackfan-Diamond anemia, HL | 18–31 (median 21) | 60% | 3–27 years (median 10.5) | 2-3 weeks after the second dose |
| Redjoul [19] | France | retrospective study | 42(allogeneic) | BNT162b2 | 3rd | Abbott | 50.0 AU/mL | NA | 59 [IQR 50–64] | 65% | NA | 26 (SD 6) days after the third dose |
| Peeters [20] | Belgium | interventional prospective multicohort study | 11(allogeneic) | BNT162b2 | 2nd | Wantai ELISA | 5 IU/ml | NA | NA | NA | NA | 28 days after the second dose |
| Chiarucci [21] | Italy | prospective study | 38(autologous)+12(allogeneic) | BNT162b2 | 2nd | DiaSorin | 15.0 AU/mL | autoHSCT: MM, NHL, HL;  alloHSCT: AML; ALL, MDS | NA | 56% | NA | 30 days after the second dose |
| Majcherek [22] | Poland | prospective study | 26(autologous)+63(allogeneic) | BNT162b2 | 2nd | Abbott | NA | AML, MDS, ALL, NHL, HL, MM and other | autoHSCT: 58 (range 26–69);  alloHSCT: 52 (range 20–68) | 53% | autoHSCT: 10 (range 4–38); alloHSCT: 23 (range 2–112) | two to four weeks after the second dose |
| Piñana [23] | Spain | prospective observational study | 86(autologous)+311(allogeneic) | BNT162b2, mRNA-1273, AZD1222, Ad26.COV2. S | 2nd or 1st | ELISA, Abbott, DiaSorin, SNIBE, Siemens | NA | AML, MDS, NHL, MM, CLL, HD, MPN, ALL and Others | autoHSCT: 64.6 (range 19–78); alloHSCT: 56.7 (range 18–80) | autoHSCT: 57%; alloHSCT: 60% | autoHSCT: 88 (range 3–763); alloHSCT: 98 (range 4–646) | autoHSCT: 22 days (range 15–52); alloHSCT: 21 days (range 15–59) |
| Salvini [24] | Italy | prospective cohort study | 64(autologous) | BNT162b2 | 2nd | DiaSorin | 33.8 BAU/ml | MM, PCL, FL, HL, MCL, MBL, DLBCL, AITL, NK/T-cell lymphoma | 62 (range 29-75) | 58% | 25.6 (range 1.2-58.1) | 28 days (range 25–48) after the second dose |
| Morsink [25] | Netherlands | retrospective study | 70(allogeneic) | BNT162b2, mRNA-1273, AZD1222 | 2nd or 1st | Abbott | 300 AU/mL | AML | NA | 59% | 27.8 (range 0.6–49.5) | 7–21 days after completed vaccination |
| Canti [26] | Belgium | a phase IV trial | 37(allogeneic) | BNT162b2 | 2nd | Wantai ELISA | 5 IU/mL | NA | 60 (range 26-76) | 48% | 31 (range 6-57) | 21 days after the second dose |
| Bergman [27] | Sweden | prospective open-label clinical trial | 72(allogeneic) | BNT162b2 | 2nd | Roche | 0.8 U/ml | NA | NA | 53% | NA | 14 days after the second dose |
| Maillard [28] | France | retrospective observational study | 687(allogeneic) | BNT162b2, mRNA-1273 | 2nd + 3rd | Abbott, Roche, DiaSorin, Siemens, ELISA | Abbott: 50 AU/mL, Roche: 0.8 U/ml, DiaSorin: 13 AU/ml, Siemens: 1.0 U/ml, Wantai ELISA: 0.75 AU/ml | Myeloid malignancy, Lymphoid malignancy, Nonmalignant | NA | 59% | 27 (IQR 14-56) | 33 days (IQR 27-52) after the second dose; 30 days (IQR 27-35) after the third dose |
| Yeshurun [29] | Israel | prospective cohort study | 106(allogeneic) | BNT162b2 | 2nd | Abbott | 50 AU/mL | Acute leukaemia, MDS, Lymphoma and others | 65 (range 23-80) | 63% | 41.5 (range 4-439) | 4-6 weeks after the second dose |
| Rahav [30] | Israel | prospective cohort study | 111(allogeneic) | BNT162b2 | 2nd | ELISA | 1.1 | NA | 62.0 (range 49.0-70.0) | 63% | 3.4 years (IQR 2.0-6.3) | 19 days (IQR 14-25) |
| Haggenburg [31] | Netherlands | prospective observational cohort study | 72(autologous)+49(allogeneic) | mRNA-1273 | 2nd | MIA | 10 BAU/ml | AutoHCT: Lymphoma, MM;  alloHCT: NA | NA | NA | 6-12 months | 28 days after the 2nd vaccination |
| Watanabe [32] | Japan | prospective observational study | 25(allogeneic) | BNT162b2 | 2nd | QuaResearch | O.D: 0.26 | AML, ALL, ML and others | 55 (range 23–71) | 56% | 53.5 (range 5.4–137.5) | 14 days (+/- 7 days) after the second dose |
| Pabst [33] | Germany | NA | 167(allogeneic) | BNT162b2, mRNA-1273, AZD1222 | 1st + 2nd | Medac | anti-S1 index ≥ 1 | AML, MDS, MPN, AA, ALL, indolent lymphoma, MM | 60 (range 19–79） | 61% | 40.5 (range 3.5–303.7) | NA |
| Debie [34] | Belgium | prospective study | 10(allogeneic) | BNT162b2 | 2nd + 3rd | ELISA | 5.4 BAU/mL | NA | NA | NA | NA | 28 days after the second/third dose |
| Nooka [35] | USA | NA | 178(autologous) | BNT162b2, mRNA-1273, AD26.COV2.S | 2nd or 1st | ELISA | 100 AU/mL | MM | NA | NA | NA | 1-2 weeks after completed vaccination |
| Beerlage [36] | Switzerland | retrospective study | 182(allogeneic) | BNT162b2, mRNA-1273 | 2nd | Roche | 1.0 U/ml | ALL, MDS/AML, Lymphoma, MPN, and others | 56 (range 21–80) | 66% | 3.25 years (range 3 months-34 years) | 51 (range 6–187) |
| Abid [37] | USA | retrospective study | 30(autologous)+26(allogeneic) | BNT162b2, mRNA-1273 | 3rd | Abbott | 50.0 AU/mL | autoHSCT: Lymphoma, Myeloma; alloHSCT: NA | autoHSCT: 67 (48-77) for seropositive patients, 62 (54-71) for seronegative patients; alloHSCT: 70 (31-75) for seropositive patients, 66 (38-78) for seronegative patients | NA | NA | 58 days (range 14-140) for seropositive patients, 47 days (range 18-127) for seronegative patients |
| Balcells [38] | Chile | prospective cohort study | 9(autologous)+5(allogeneic) | CoronaVac | 2nd | Euroimmun ELASA | 11 RU/ml | NA | 47.4 (range 49.0) | 71% | NA | 8-12 weeks after the second |
| Tsushima [39] | Japan | retrospective study | 65(autologous)+65(allogeneic) | BNT162b2, mRNA-1273 | 2nd | Roche | 0.8 U/ml | autoHSCT: NHL, HL, MM; alloHSCT: AML, MDS, CML, ALL, NHL, HL, MM, AA | autoHSCT: 70 (34–79); alloHSCT: 55 (23–80) | autoHSCT: 51; alloHSCT: 63 | autoHSCT: 49 (4 -185); alloHSCT: 92 (4–255) | three to eight weeks after the second dose |
| Huang [40] | Switzerland | prospective observational study | 110(allogeneic) | BNT162b2, mRNA-1273 | 1st+2nd | Roche | 0.8 U/ml | Myeloid, Lymphoid, and others | 57 (IQR 46-65) | 63% | 20 months (range 3 months-35 years) | before the second dose and 2 to 6 weeks after the second dose |
| Einarsdottir [41] | Sweden | NA | 50(allogeneic) | BNT162b2, mRNA-1273 | 1st+2nd | Abbott | 50.0 AU/mL | AML, ALL, CML, CLL, Lymphoma, MDS, MF, AA, MM, Thalassemia | 54 (range 29-78) | 52% | 92 (range 7-340) | 27 days (range 16-33) after the second dose |
| Einarsdottir [42] | Sweden | NA | 37(allogeneic) | BNT162b2, mRNA-1273 | 3rd | Abbott | 50.0 AU/mL(14 BAU/mL) | AML, ALL, MDS, MF, CML, atypical CML, myeloma, CLL, Hodgkin’s disease, STAT-1 immune deficiency | positive: 60 (range 19-78); negative: 63 (range 32-72) | NA | 23 (range 6-191) | 24 days (range 19-30) after the third dose |
| Canti [43] | Belgium | NA | 38(allogeneic) | BNT162b2 | 3rd | Wantai | 5 BAU/mL | NA | 60 (range 26-76) | 50% | 31 (range 6-58) | 28 days after the third dose |
| Chang [44] | USA | NA | 15(autologous) | BNT162b2, mRNA-1273 | 2nd | Mesoscale Discovery | NA | NA | NA | NA | NA | NA |

*: including 7 patients of CAR-T; NA: not available; IQR: interquartile range; HSCT: hematopoietic stem cell transplantation; autoHSCT: autologous HSCT; alloHSCT: allogeneic HSCT; Mixed: containing patients of autologous/allogeneic HSCT; ALL: Acute lymphoblastic leukemia; AML: Acute myeloid leukemia; HLH: Hemophagocytic lymphohistiocytosis; CML: Chronic myeloid leukemia; HL: Hodgkin lymphoma; AA: Aplastic anemia; MM: Multiple myeloma; FL: Follicular lymphoma; DLBCL: Diffuse Large B-Cell Lymphoma; LBCL: Large B-cell lymphoma; CLL: Chronic lymphocytic leukemia; MCL: Mantle cell lymphoma; T-NHL: T-Cell non-Hodgkin lymphoma; B-NHL: B-Cell Non-Hodgkin lymphoma; MDS: Myelodysplastic syndrome; NHL: Non-Hodgkin lymphoma; MF: Myelofibrosis; MPD: Myeloproliferative disorders; SAA: Severe aplastic anaemia; CMML: Chronic myelomonocytic leukaemia; MPN: Myeloproliferative neoplasm; PCL: Plasma cell leukemia; MBL: Mediastinal B-cell lymphoma; AITL: Angioimmunoblastic T-cell lymphoma; ML: Malignant lymphoma;

1st: studies evaluating immunological response after one dose of mRNA vaccine;

2nd: studies evaluating immunological response after completed vaccination (two dose of mRNA vaccines or inactivated vaccines);

3rd: studies evaluating immunological response after the third dose of mRNA vaccine;

1st + 2nd: studies evaluating immunological response both after first and second vaccination;

2nd + 3rd: studies evaluating immunological response both after second and third vaccination;

2nd or 1st: studies evaluating immunological response after completed vaccination (two doses of mRNA/inactivated vaccines or one dose of adenoviral vaccine).

**Supplementary Table S2. Study characteristics for CAR-T.**

| Study | Country | Study design | No. of patients | Vaccine type | Dosage | Test assay | Cut-off | Disease | Median age, years (range) | Gender (male %) | Median time for vaccination after CAR-T, months (range) | Internal between the vaccine and response assessment | CAR-T construct |
| --- | --- | --- | --- | --- | --- | --- | --- | --- | --- | --- | --- | --- | --- |
| Dhakal [3] | USA | NA | 14 | BNT162b2, mRNA-1273, Ad26.COV2.S | 2nd or 1st | Euroimmun ELISA | 1.1 | NA | NA | NA | 24 (8–31) | ≥ 2weeks after completed vaccination | NA |
| Tamari [4] | USA | prospective observational study | 7 | BNT162b2, mRNA-1273 | 2nd | Abbott | 50.0 AU/mL | NA | NA | NA | NA | about 2 months after the second dose | NA |
| Ram [9] | Israel | prospective cohort study | 14 | BNT162b2 | 2nd | Roche | 0.8 U/ml | NA | NA | NA | 9 (3-17) | 7-14 days after the second dose | CD19 |
| Greenberger [10] | USA | prospective cohort study | 12 | BNT162b2, mRNA-1273 | 2nd | Roche | 0.8 U/ml | DLBCL, CLL, FL, MM | NA | NA | NA | 41 days (14, 134) after the second dose | CD19 (n = 7); BCMA‐ or CD138 (n = 5) |
| Thakkar [11] | USA | cross-sectional cohort study | 3 | BNT162b2, mRNA-1273, AD26.COV2.S | 2nd or 1st | Abbott | 50.0 AU/mL | NA | NA | NA | NA | at least 7 days after the 2nd of the vaccine | NA |
| Van Oekelen [12] | USA | NA | 19 | BNT162b2, mRNA-1273 | 2nd | Kantaro ELISA | 5 AU/mL | MM | 68 (38-93) | NA | NA | 51 days (range 11–118) after the second dose | NA |
| Bergman [27] | Sweden | prospective open-label clinical trial | 2 | BNT162b2 | 2nd | Roche | 0.8 U/ml | NA | 67 | NA | NA | 14 days after the second dose | CD19 |
| Haggenburg [31] | Netherlands | prospective observational cohort study | 44 | mRNA-1273 | 2nd | MIA | 10 BAU/ml | NA | NA | NA | NA | 28 days after the 2nd vaccination | CD19 |
| Gastinne [45] | France | NA | 20 | BNT162b2 | 2nd | Roche | NA | B‐NHL, ALL | 62 (21-79) | 61% | 13 (4–27) | 52 days (range 21–99) after the second dose | NA |
| Dahiya [46] | USA | prospective study | 14 | BNT162b2, mRNA-1273 | 2nd | Invitrogen ELISA | NA | LBCL, MCL, FL | NA | 67% | NA | 4 weeks after the second dose of the vaccine (14 patients), 4 weeks after the first dose (4 patients) | CD19 |
| Nooka [35] | USA | NA | 15 | BNT162b2, mRNA-1273, Ad26.COV2.S | 2nd or 1st | ELISA | 100 AU/mL | MM | NA | NA | NA | 1-2 weeks after the second dose | NA |
| Abid [37] | USA | retrospective study | 10 | BNT162b2, mRNA-1273 | 3rd | Abbott | 50 AU/mL | NA | 72 (70-75) for seropositive patients; 68 (35-81) for seronegative patients | NA | NA | 58 days (range 14-140) for seropositive patients, 47 days (range 18-127) for seronegative patients | CD19+BCMA |

NA: not available; CAR-T: chimeric antigen receptor T-cell; DLBCL: Diffuse Large B-Cell Lymphoma; CLL: Chronic lymphocytic leukemia; FL: Follicular lymphoma; MM: Multiple myeloma; B-NHL: B-Cell Non-Hodgkin lymphoma; ALL: Acute lymphoblastic leukemia; LBCL: Large B-cell lymphoma; MCL: Mantle cell lymphoma.

**Supplemental Table S3. Anti-Spike (S) SARS-CoV-2 antibody titres in HSCT patients vs. healthy controls.**

| study | Dose | HSCT type | HSCT | | | Healthy controls | | | *P-*value |
| --- | --- | --- | --- | --- | --- | --- | --- | --- | --- |
|  |  |  | **n** | **Median** | **IQR/range/CI** | **n** | **Median** | **IQR/range /CI** |  |
| Tamari | 2 | allogeneic | 149 | 5019.8 | 635.4-15914.5 | 54 | 7720 | 3885-9746 | 0.024 |
| Maneikis | 2 | allogeneic | 122 | 6304 | 1120-16913 | 68 | 21395 | 14831-33553 | <0.0001 |
| Shem-Tov | 2 | allogeneic | 152 | 2.61 | CI 2.16-3.16 | 272 | 5.98 | CI 5.70-6.28 | <0.0001 |
| Attolico | 2 | allogeneic | 62 | 6576 | range 0-77673 | 107 | 7132 | range 217-67282 | NS |
| Rahav | 2 | allogeneic | 111 | 2.55 | CI 2.03-3.21 | 272 | 5.98 | CI 5.70-6.28 | <0.0001 |
| Tsushima | 2 | allogeneic | 65 | 900 | range 0.4-12893 | 140 | 669 | 40.3-4377 | <0.001 |
| Maneikis | 2 | autologous | 192 | 6203 | 1451-16834 | 68 | 21395 | 14831-33553 | <0.0001 |
| Tsushima | 2 | autologous | 65 | 178 | range 0.4-4990 | 140 | 669 | 40.3-4377 | <0.001 |
| Herzog Tzarfati | 2 | autologous | 21 | 95.4 | 10.4-214 | 108 | 157 | 130-221 | <0.001 |
| Attolico | 2 | autologous | 52 | 4023 | range 1.3-104689 | 107 | 7132 | range 217-67282 | 0.04 |
| Tamari | 2 | autologous | 61 | 2260.1 | 419.4-10598.8 | 54 | 7720 | 3885-9746 | <0.001 |

NS: no significance; IQR: interquartile range; CI: 95% confidence interval.

**References:**

[1] Herzog Tzarfati K, Gutwein O, Apel A, Rahimi-Levene N, Sadovnik M, Harel L, Benveniste-Levkovitz P, Bar Chaim A, Koren-Michowitz M. BNT162b2 COVID-19 vaccine is significantly less effective in patients with hematologic malignancies. Am J Hematol. 2021 Oct 1;96(10):1195-1203. doi: 10.1002/ajh.26284.

[2] Avivi I, Balaban R, Shragai T, Sheffer G, Morales M, Aharon A, Lowenton-Spier N, Trestman S, Perry C, Benyamini N, Mittelman M, Tabib Y, Bar Lev T, Zavaro M, Herishanu Y, Luttwak E, Cohen YC. Humoral response rate and predictors of response to BNT162b2 mRNA COVID19 vaccine in patients with multiple myeloma. Br J Haematol. 2021 Oct;195(2):186-193. doi: 10.1111/bjh.17608.

[3] Dhakal B, Abedin S, Fenske T, Chhabra S, Ledeboer N, Hari P, Hamadani M. Response to SARS-CoV-2 vaccination in patients after hematopoietic cell transplantation and CAR T-cell therapy. Blood. 2021 Oct 7;138(14):1278-1281. doi: 10.1182/blood.2021012769.

[4] Tamari R, Politikos I, Knorr DA, Vardhana SA, Young JC, Marcello LT, Doddi S, Devlin SM, Ramanathan LV, Pessin MS, Dunn E, Palazzo M, Bravo CD, Papanicolaou GA, Kamboj M, Perales MA, Chung DJ, Shah GL. Predictors of Humoral Response to SARS-CoV-2 Vaccination after Hematopoietic Cell Transplantation and CAR T-cell Therapy. Blood Cancer Discov. 2021 Sep 13;2(6):577-585. doi: 10.1158/2643-3230.BCD-21-0142.

[5] Le Bourgeois A, Coste-Burel M, Guillaume T, Peterlin P, Garnier A, Béné MC, Chevallier P. Safety and Antibody Response After 1 and 2 Doses of BNT162b2 mRNA Vaccine in Recipients of Allogeneic Hematopoietic Stem Cell Transplant. JAMA Netw Open. 2021 Sep 1;4(9):e2126344. doi: 10.1001/jamanetworkopen.2021.26344.

[6] Maneikis K, Šablauskas K, Ringelevičiūtė U, Vaitekėnaitė V, Čekauskienė R, Kryžauskaitė L, Naumovas D, Banys V, Pečeliūnas V, Beinortas T, Griškevičius L. Immunogenicity of the BNT162b2 COVID-19 mRNA vaccine and early clinical outcomes in patients with haematological malignancies in Lithuania: a national prospective cohort study. Lancet Haematol. 2021 Aug;8(8):e583-e592. doi: 10.1016/S2352-3026(21)00169-1.

[7] Easdale S, Shea R, Ellis L, Bazin J, Davis K, Dallas F, Thistlethwayte E, Ethell M, Potter M, Arias C, Anthias C, Nicholson E. Serologic Responses following a Single Dose of SARS-Cov-2 Vaccination in Allogeneic Stem Cell Transplantation Recipients. Transplant Cell Ther. 2021 Oct;27(10):880.e1-880.e4. doi: 10.1016/j.jtct.2021.07.011.

[8] Redjoul R, Le Bouter A, Beckerich F, Fourati S, Maury S. Antibody response after second BNT162b2 dose in allogeneic HSCT recipients. Lancet. 2021 Jul 24;398(10297):298-299. doi: 10.1016/S0140-6736(21)01594-4.

[9] Ram R, Hagin D, Kikozashvilli N, Freund T, Amit O, Bar-On Y, Beyar-Katz O, Shefer G, Moshiashvili MM, Karni C, Gold R, Kay S, Glait-Santar C, Eshel R, Perry C, Avivi I, Apel A, Benyamini N, Shasha D, Ben-Ami R. Safety and Immunogenicity of the BNT162b2 mRNA COVID-19 Vaccine in Patients after Allogeneic HCT or CD19-based CART therapy-A Single-Center Prospective Cohort Study. Transplant Cell Ther. 2021 Sep;27(9):788-794. doi: 10.1016/j.jtct.2021.06.024.

[10] Greenberger LM, Saltzman LA, Senefeld JW, Johnson PW, DeGennaro LJ, Nichols GL. Antibody response to SARS-CoV-2 vaccines in patients with hematologic malignancies. Cancer Cell. 2021 Aug 9;39(8):1031-1033. doi: 10.1016/j.ccell.2021.07.012.

[11] Thakkar A, Gonzalez-Lugo JD, Goradia N, Gali R, Shapiro LC, Pradhan K, Rahman S, Kim SY, Ko B, Sica RA, Kornblum N, Bachier-Rodriguez L, McCort M, Goel S, Perez-Soler R, Packer S, Sparano J, Gartrell B, Makower D, Goldstein YD, Wolgast L, Verma A, Halmos B. Seroconversion rates following COVID-19 vaccination among patients with cancer. Cancer Cell. 2021 Aug 9;39(8):1081-1090.e2. doi: 10.1016/j.ccell.2021.06.002.

[12] Van Oekelen O, Gleason CR, Agte S, Srivastava K, Beach KF, Aleman A, Kappes K; PVI/Seronet team, Mouhieddine TH, Wang B, Chari A, Cordon-Cardo C, Krammer F, Jagannath S, Simon V, Wajnberg A, Parekh S. Highly variable SARS-CoV-2 spike antibody responses to two doses of COVID-19 RNA vaccination in patients with multiple myeloma. Cancer Cell. 2021 Aug 9;39(8):1028-1030. doi: 10.1016/j.ccell.2021.06.014.

[13] Ehmsen S, Asmussen A, Jeppesen SS, Nilsson AC, Østerlev S, Vestergaard H, Justesen US, Johansen IS, Frederiksen H, Ditzel HJ. Antibody and T cell immune responses following mRNA COVID-19 vaccination in patients with cancer. Cancer Cell. 2021 Aug 9;39(8):1034-1036. doi: 10.1016/j.ccell.2021.07.016.

[14] Shem-Tov N, Yerushalmi R, Danylesko I, Litachevsky V, Levy I, Olmer L, Lusitg Y, Avigdor A, Nagler A, Shimoni A, Rahav G. Immunogenicity and safety of the BNT162b2 mRNA COVID-19 vaccine in haematopoietic stem cell transplantation recipients. Br J Haematol. 2022 Feb;196(4):884-891. doi: 10.1111/bjh.17918.

[15] Attolico I, Tarantini F, Carluccio P, Schifone CP, Delia M, Gagliardi VP, Perrone T, Gaudio F, Longo C, Giordano A, Sgherza N, Curci P, Rizzi R, Ricco A, Russo Rossi A, Albano F, Larocca AMV, Vimercati L, Tafuri S, Musto P. Serological response following BNT162b2 anti-SARS-CoV-2 mRNA vaccination in haematopoietic stem cell transplantation patients. Br J Haematol. 2022 Feb;196(4):928-931. doi: 10.1111/bjh.17873.

[16] Le Bourgeois A, Coste-Burel M, Guillaume T, Peterlin P, Garnier A, Imbert BM, Drumel T, Mahé B, Dubruille V, Blin N, Lok A, Touzeau C, Gastinne T, Tessoulin B, Jullien M, Vantyghem S, Moreau P, Le Gouill S, Béné MC, Chevallier P. Interest of a third dose of BNT162b2 anti-SARS-CoV-2 messenger RNA vaccine after allotransplant. Br J Haematol. 2022 Mar;196(5):e38-e40. doi: 10.1111/bjh.17911.

[17] Lindemann M, Klisanin V, Thümmler L, Fisenkci N, Tsachakis-Mück N, Ditschkowski M, Schwarzkopf S, Klump H, Reinhardt HC, Horn PA, Koldehoff M. Humoral and Cellular Vaccination Responses against SARS-CoV-2 in Hematopoietic Stem Cell Transplant Recipients. Vaccines (Basel). 2021 Sep 25;9(10):1075. doi: 10.3390/vaccines9101075.

[18] Matkowska-Kocjan A, Owoc-Lempach J, Chruszcz J, Kuźnik E, Szenborn F, Jurczenko L, Wójcik M, Banyś D, Szenborn L, Ussowicz M. The COVID-19 mRNA BNT163b2 Vaccine Was Well Tolerated and Highly Immunogenic in Young Adults in Long Follow-Up after Haematopoietic Stem Cell Transplantation. Vaccines (Basel). 2021 Oct 19;9(10):1209. doi: 10.3390/vaccines9101209.

[19] Redjoul R, Le Bouter A, Parinet V, Fourati S, Maury S. Antibody response after third BNT162b2 dose in recipients of allogeneic HSCT. Lancet Haematol. 2021 Oct;8(10):e681-e683. doi: 10.1016/S2352-3026(21)00274-X.

[20] Peeters M, Verbruggen L, Teuwen L, Vanhoutte G, Vande Kerckhove S, Peeters B, Raats S, Van der Massen I, De Keersmaecker S, Debie Y, Huizing M, Pannus P, Neven K, Ariën KK, Martens GA, Van Den Bulcke M, Roelant E, Desombere I, Anguille S, Goossens M, Vandamme T, van Dam P. Reduced humoral immune response after BNT162b2 coronavirus disease 2019 messenger RNA vaccination in cancer patients under antineoplastic treatment. ESMO Open. 2021 Oct;6(5):100274. doi: 10.1016/j.esmoop.2021.100274.

[21] Chiarucci M, Paolasini S, Isidori A, Guiducci B, Loscocco F, Capalbo M, Visani G. Immunological Response Against SARS-COV-2 After BNT162b2 Vaccine Administration Is Impaired in Allogeneic but Not in Autologous Stem Cell Transplant Recipients. Front Oncol. 2021 Sep 6;11:737300. doi: 10.3389/fonc.2021.737300.

[22] Majcherek M, Matkowska-Kocjan A, Szymczak D, Karasek M, Szeremet A, Kiraga A, Milanowska A, Kuznik E, Kujawa K, Wrobel T, Szenborn L, Czyz A. Two Doses of BNT162b2 mRNA Vaccine in Patients after Hematopoietic Stem Cell Transplantation: Humoral Response and Serological Conversion Predictors. Cancers (Basel). 2022 Jan 10;14(2):325. doi: 10.3390/cancers14020325.

[23] Piñana JL, López-Corral L, Martino R, Montoro J, Vazquez L, Pérez A, Martin-Martin G, Facal-Malvar A, Ferrer E, Pascual MJ, Sanz-Linares G, Gago B, Sanchez-Salinas A, Villalon L, Conesa-Garcia V, Olave MT, López-Jimenez J, Marcos-Corrales S, García-Blázquez M, Garcia-Gutiérrez V, Hernández-Rivas JÁ, Saus A, Espigado I, Alonso C, Hernani R, Solano C, Ferrer-Lores B, Guerreiro M, Ruiz-García M, Muñoz-Bellido JL, Navarro D, Cedillo A, Sureda A; Infectious Complications Subcommittee of the Spanish Hematopoietic Stem Cell Transplantation and Cell Therapy Group (GETH-TC). SARS-CoV-2-reactive antibody detection after SARS-CoV-2 vaccination in hematopoietic stem cell transplant recipients: Prospective survey from the Spanish Hematopoietic Stem Cell Transplantation and Cell Therapy Group. Am J Hematol. 2022 Jan 1;97(1):30-42. doi: 10.1002/ajh.26385.

[24] Salvini M, Maggi F, Damonte C, Mortara L, Bruno A, Mora B, Brociner M, Mattarucchi R, Ingrassia A, Sirocchi D, Bianchi B, Agnoli S, Gallazzi M, Merli M, Ferrario A, Bombelli R, Barraco D, Baj A, Bertù L, Grossi PA, Passamonti F. Immunogenicity of anti-SARS-CoV-2 Comirnaty vaccine in patients with lymphomas and myeloma who underwent autologous stem cell transplantation. Bone Marrow Transplant. 2022 Jan;57(1):137-139. doi: 10.1038/s41409-021-01487-4.

[25] Morsink LM, van Doesum J, Choi G, Hazenberg CLE, Biswana A, Meppelink F, Bungener LB, Lambeck AJA, Huls G. Robust COVID-19 vaccination response after allogeneic stem cell transplantation using post transplantation cyclophosphamide conditioning. Blood Cancer J. 2022 Jan 12;12(1):6. doi: 10.1038/s41408-021-00605-1.

[26] Canti L, Humblet-Baron S, Desombere I, Neumann J, Pannus P, Heyndrickx L, Henry A, Servais S, Willems E, Ehx G, Goriely S, Seidel L, Michiels J, Willems B, Liston A, Ariën KK, Beguin Y, Goossens ME, Marchant A, Baron F. Predictors of neutralizing antibody response to BNT162b2 vaccination in allogeneic hematopoietic stem cell transplant recipients. J Hematol Oncol. 2021 Oct 24;14(1):174. doi: 10.1186/s13045-021-01190-3.

[27] Bergman P, Blennow O, Hansson L, Mielke S, Nowak P, Chen P, Söderdahl G, Österborg A, Smith CIE, Wullimann D, Vesterbacka J, Lindgren G, Blixt L, Friman G, Wahren-Borgström E, Nordlander A, Gomez AC, Akber M, Valentini D, Norlin AC, Thalme A, Bogdanovic G, Muschiol S, Nilsson P, Hober S, Loré K, Chen MS, Buggert M, Ljunggren HG, Ljungman P, Aleman S; COVAXID-collaborator group (shown separately). Safety and efficacy of the mRNA BNT162b2 vaccine against SARS-CoV-2 in five groups of immunocompromised patients and healthy controls in a prospective open-label clinical trial. EBioMedicine. 2021 Dec;74:103705. doi: 10.1016/j.ebiom.2021.103705.

[28] Maillard A, Redjoul R, Klemencie M, Labussière Wallet H, Le Bourgeois A, D'Aveni M, Huynh A, Berceanu A, Marchand T, Chantepie S, Botella Garcia C, Loschi M, Joris M, Castilla-Llorente C, Thiebaut-Bertrand A, François S, Leclerc M, Chevallier P, Nguyen S. Antibody response after 2 and 3 doses of SARS-CoV-2 mRNA vaccine in allogeneic hematopoietic cell transplant recipients. Blood. 2022 Jan 6;139(1):134-137. doi: 10.1182/blood.2021014232.

[29] Yeshurun M, Pasvolsky O, Shargian L, Yahav D, Ben-Zvi H, Rubinstein M, Sela-Navon M, Wolach O, Raanani P, Rozovski U. Humoral serological response to the BNT162b2 vaccine after allogeneic haematopoietic cell transplantation. Clin Microbiol Infect. 2022 Feb;28(2):303.e1-303.e4. doi: 10.1016/j.cmi.2021.10.007.

[30] Rahav G, Lustig Y, Lavee J, Ohad Benjamini, Magen H, Hod T, Noga Shem-Tov, Shmueli ES, Drorit Merkel, Ben-Ari Z, Halperin R, Indenbaum V, Olmer L, Huppert A, Mor E, Regev-Yochay G, Cohen C, Finesod AW, Levy I. BNT162b2 mRNA COVID-19 vaccination in immunocompromised patients: A prospective cohort study. EClinicalMedicine. 2021 Nov;41:101158. doi: 10.1016/j.eclinm.2021.101158.

[31] Haggenburg S, Lissenberg-Witte BI, van Binnendijk RS, den Hartog G, Bhoekhan MS, Haverkate NJE, de Rooij DM, van Meerloo J, Cloos J, Kootstra NA, Wouters D, Weijers SS, van Leeuwen EMM, Bontkes HJ, Tonouh-Aajoud S, Heemskerk MHM, Sanders RW, Roelandse-Koop E, Hofsink Q, Groen K, Çetinel L, Schellekens L, den Hartog YM, Toussaint B, Kant IMJ, Graas T, de Pater E, Dik WA, Engel MD, Pierie CRN, Janssen SR, van Dijkman E, Poniman M, Burger JA, Bouhuijs JH, Smits G, Rots NY, Zweegman S, Kater AP, van Meerten T, Mutsaers PGNJ, van Doesum JA, Broers AEC, van Gils MJ, Goorhuis A, Rutten CE, Hazenberg MD, Nijhof IS. Quantitative analysis of mRNA-1273 COVID-19 vaccination response in immunocompromised adult hematology patients. Blood Adv. 2022 Mar 8;6(5):1537-1546. doi: 10.1182/bloodadvances.2021006917.

[32] Watanabe M, Yakushijin K, Funakoshi Y, Ohji G, Hojo W, Sakai H, Saeki M, Hirakawa Y, Matsumoto S, Sakai R, Nagao S, Kitao A, Miyata Y, Koyama T, Saito Y, Kawamoto S, Ito M, Murayama T, Matsuoka H, Minami H. The Safety and Immunogenicity of the BNT162b2 mRNA COVID-19 Vaccine in Japanese Patients after Allogeneic Stem Cell Transplantation. Vaccines (Basel). 2022 Jan 21;10(2):158. doi: 10.3390/vaccines10020158.

[33] Pabst C, Benning L, Liebers N, Janssen M, Caille L, Speer C, He L, Schubert ML, Simons L, Hegenbart U, Schönland S, Radujkovic A, Schmitt M, Schnitzler P, Müller-Tidow C, Dietrich S, Dreger P, Luft T. Humoral Responses and Chronic GVHD Exacerbation after COVID-19 Vaccination Post Allogeneic Stem Cell Transplantation. Vaccines (Basel). 2022 Feb 18;10(2):330. doi: 10.3390/vaccines10020330.

[34] Debie Y, Vandamme T, Goossens ME, van Dam PA, Peeters M. Antibody titres before and after a third dose of the SARS-CoV-2 BNT162b2 vaccine in patients with cancer. Eur J Cancer. 2022 Mar;163:177-179. doi: 10.1016/j.ejca.2021.12.025.

[35] Nooka AK, Shanmugasundaram U, Cheedarla N, Verkerke H, Edara VV, Valanparambil R, Kaufman JL, Hofmeister CC, Joseph NS, Lonial S, Azeem M, Manalo J, Switchenko JM, Chang A, Linderman SL, Roback JD, Dhodapkar KM, Ahmed R, Suthar MS, Neish AS, Dhodapkar MV. Determinants of Neutralizing Antibody Response After SARS CoV-2 Vaccination in Patients With Myeloma. J Clin Oncol. 2022 Mar 8:JCO2102257. doi: 10.1200/JCO.21.02257.

[36] Beerlage A, Leuzinger K, Valore L, Mathew R, Junker T, Drexler B, Passweg JR, Hirsch HH, Halter J. Antibody response to mRNA SARS-CoV-2 vaccination in 182 patients after allogeneic hematopoietic cell transplantation. Transpl Infect Dis. 2022 Mar 24:e13828. doi: 10.1111/tid.13828.

[37] Abid MB, Rubin M, Ledeboer N, Szabo A, Longo W, Mohan M, Shah NN, Fenske TS, Abedin S, Runaas L, D'Souza A, Chhabra S, Dhakal B, Hamadani M. Efficacy of a third SARS-CoV-2 mRNA vaccine dose among hematopoietic cell transplantation, CAR T cell, and BiTE recipients. Cancer Cell. 2022 Apr 11;40(4):340-342. doi: 10.1016/j.ccell.2022.02.010.

[38] Balcells ME, Le Corre N, Durán J, Ceballos ME, Vizcaya C, Mondaca S, Dib M, Rabagliati R, Sarmiento M, Burgos PI, Espinoza M, Ferrés M, Martinez-Valdebenito C, Ruiz-Tagle C, Ortiz C, Ross P, Budnik S, Solari S, Vizcaya MLÁ, Lembach H, Berrios-Rojas R, Melo-González F, Ríos M, Kalergis AM, Bueno SM, Nervi B. Reduced immune response to inactivated SARS-CoV-2 vaccine in a cohort of immunocompromised patients in Chile. Clin Infect Dis. 2022 Mar 7:ciac167. doi: 10.1093/cid/ciac167.

[39] Tsushima T, Terao T, Narita K, Fukumoto A, Ikeda D, Kamura Y, Kuzume A, Tabata R, Miura D, Takeuchi M, Matsue K. Antibody response to COVID-19 vaccine in 130 recipients of hematopoietic stem cell transplantation. Int J Hematol. 2022 May;115(5):611-615. doi: 10.1007/s12185-022-03325-9.

[40] Huang A, Cicin-Sain C, Pasin C, Epp S, Audigé A, Müller NJ, Nilsson J, Bankova A, Wolfensberger N, Vilinovszki O, Nair G, Hockl P, Schanz U, Kouyos RD, Hasse B, Zinkernagel AS, Trkola A, Manz MG, Abela IA, Müller AMS. Antibody Response to SARS-CoV-2 Vaccination in Patients following Allogeneic Hematopoietic Cell Transplantation. Transplant Cell Ther. 2022 Apr;28(4):214.e1-214.e11. doi: 10.1016/j.jtct.2022.01.019.

[41] Einarsdottir S, Martner A, Waldenström J, Nicklasson M, Ringlander J, Arabpour M, Törnell A, Wiktorin HG, Nilsson S, Bittar R, Nilsson M, Lisak M, Veje M, Friman V, Al-Dury S, Bergström T, Ljungman P, Brune M, Hellstrand K, Lagging M. Deficiency of SARS-CoV-2 T-cell responses after vaccination in long-term allo-HSCT survivors translates into abated humoral immunity. Blood Adv. 2022 May 10;6(9):2723-2730. doi: 10.1182/bloodadvances.2021006937.

[42] Einarsdottir S, Martner A, Nicklasson M, Wiktorin HG, Arabpour M, Törnell A, Vaht K, Waldenström J, Ringlander J, Bergström T, Brune M, Hellstrand K, Ljungman P, Lagging M. Reduced immunogenicity of a third COVID-19 vaccination among recipients of allogeneic haematopoietic stem cell transplantation. Haematologica. 2022 Mar 3. doi: 10.3324/haematol.2021.280494.

[43] Canti L, Ariën KK, Desombere I, Humblet-Baron S, Pannus P, Heyndrickx L, Henry A, Servais S, Willems E, Ehx G, Goriely S, Seidel L, Michiels J, Willems B, Goossens ME, Beguin Y, Marchant A, Baron F. Antibody response against SARS-CoV-2 Delta and Omicron variants after third-dose BNT162b2 vaccination in allo-HCT recipients. Cancer Cell. 2022 Apr 11;40(4):335-337. doi: 10.1016/j.ccell.2022.02.005.

[44] Chang A, Akhtar A, Linderman SL, Lai L, Orellana-Noia VM, Valanparambil R, Ahmed H, Zarnitsyna VI, McCook-Veal AA, Switchenko JM, Koff JL, Blum KA, Ayers AA, O'Leary CB, Churnetski MC, Sulaiman S, Kives M, Sheng P, Davis CW, Nooka AK, Antia R, Dhodapkar MV, Suthar MS, Cohen JB, Ahmed R. Humoral Responses Against SARS-CoV-2 and Variants of Concern After mRNA Vaccines in Patients With Non-Hodgkin Lymphoma and Chronic Lymphocytic Leukemia. J Clin Oncol. 2022 Apr 18:JCO2200088. doi: 10.1200/JCO.22.00088.

[45] Gastinne T, Le Bourgeois A, Coste-Burel M, Guillaume T, Peterlin P, Garnier A, Imbert BM, Drumel T, Mahe B, Dubruille V, Blin N, Lok A, Touzeau C, Tessoulin B, Jullien M, Vanthygem S, Béné MC, Moreau P, Le Gouill S, Chevallier P. Safety and antibody response after one and/or two doses of BNT162b2 Anti-SARS-CoV-2 mRNA vaccine in patients treated by CAR T cells therapy. Br J Haematol. 2022 Jan;196(2):360-362. doi: 10.1111/bjh.17818.

[46] Dahiya S, Luetkens T, Lutfi F, Avila S, Iraguha T, Margiotta P, Hankey KG, Lesho P, Law JY, Lee ST, Baddley J, Kocoglu M, Yared JA, Hardy NM, Rapoport AP, Atanackovic D. Impaired immune response to COVID-19 vaccination in patients with B-cell malignancies after CD19 CAR T-cell therapy. Blood Adv. 2022 Jan 25;6(2):686-689. doi: 10.1182/bloodadvances.2021006112.
